# A Standardized Framework for Evaluating Surgical Enhanced Recovery Pathways: A Recommendations Statement from the TDABC in Health-care Consortium

**DOI:** 10.36469/001c.24590

**Published:** 2021-06-24

**Authors:** Ana Paula B.S. Etges, Luciana Paula Cadore Stefani, Dionisios Vrochides, Junaid Nabi, Carisi Anne Polanczyk, Richard D. Urman

**Affiliations:** 1 National Institute of Science and Technology for Health Technology Assessment (IATS) - CNPq/Brazil (project: 465518/2014-1), Porto Alegre, RS, Brazil; School of Technology, Pontifícia Universidade Católica do Rio Grande do Sul, Porto Alegre, Brazil; Postgraduate Program in Epidemiology, Universidade Federal do Rio Grande do Sul, Porto Alegre, Brazil; 2 Department of Surgery Hospital de Clínicas de Porto Alegre, Universidade Federal do Rio Grande do Sul, Porto Alegre, RS, Brazil; 3 Department of Surgery Division of Hepatobiliary & Pancreas Surgery, Carolinas Medical Center, Atrium Health, Charlotte, NC, USA; 4 Harvard University, Harvard Business School, Boston, MA, USA; 5 National Institute of Science and Technology for Health Technology Assessment (IATS) - CNPq/Brazil (project: 465518/2014-1), Porto Alegre, RS, Brazil; Postgraduate Program in Epidemiology, Universidade Federal do Rio Grande do Sul, Porto Alegre, Brazil; 6 Department of Anesthesiology, Perioperative and Pain Medicine, Brigham and Women’s Hospital/Harvard Medical School

**Keywords:** value-based health care, enhanced recovery pathway, time-driven activity-based costing

## Abstract

**Background:** Innovative methodologies to redesign care delivery are being applied to increase value in health care, including the creation of enhanced recovery pathways (ERPs) for surgical patients. However, there is a lack of standardized methods to evaluate ERP implementation costs.

**Objectives:** This Recommendations Statement aims to introduce a standardized framework to guide the economic evaluation of ERP care-design initiatives, using the Time-Driven Activity-Based Costing (TDABC) methodology.

**Methods:** We provide recommendations on using the proposed framework to support the decision-making processes that incorporate ERPs. Since ERPs are usually composed of activities distributed throughout the patient care pathway, the framework can demonstrate how the TDABC may be a valuable method to evaluate the incremental costs of protocol implementation. Our recommendations are based on the review of available literature and expert opinions of the members of the TDABC in Healthcare Consortium.

**Results:** The ERP framework, composed of 11 steps, was created describing how the techniques and methods can be applied to evaluate the economic impact of an ERP and guide health-care leaders to optimize the decision-making process of incorporating ERPs into health-care settings. Finally, six recommendations are introduced to demonstrate that using the suggested framework could increase value in ERP care-design initiatives by reducing variability in care delivery, educating multidisciplinary teams about value in health, and increasing transparency when managing surgical pathways.

**Conclusions:** Our proposed standardized framework can guide decisions and support measuring improvements in value achieved by incorporating the perioperative redesign protocols.

## INTRODUCTION

Health-care systems continuously register a significant waste of scarce resources.^1^ These worrying trends necessitate the development of innovative methodologies to redesign care delivery. The creation of enhanced recovery pathways (ERPs) for surgical patients has significantly improved care, leading to better outcomes, decreased health-care costs, and increased patient satisfaction.^2,3^ The idea behind ERPs is a series of patient interventions for surgical patients during preoperative, intraoperative, and postoperative periods to improve patient recovery and decrease complications.^3,4^ Clinical pathways emphasize preoperative optimization, multimodal analgesia, nutritional support, minimally invasive surgery, better prevention of anesthesia side effects and surgical complications, and postoperative physical activity.^4^ ERP guidelines, such as those designed for colorectal and gynecologic surgery, generally outline a comprehensive care design of the patient’s journey along a single, specific operative pathway,^5^ including several concepts from integrated practice units suggested in the value agenda introduced by Porter and Teisberg.^6^ Redesigning surgical pathways using ERP principles requires investments in technology; more importantly, it demands improvements in internal hospital practices.^7^

Evidence demonstrates that due to their impact on patient clinical outcomes (health status achieved or retained; the process of recovery; and sustainability of health), ERP initiatives have contributed to decreased overall costs of care and fewer complications.^5,8^ At the same time, the evaluation of the impact of these redesign projects still lacks standardized methods to assess all-important clinical and economic outcomes and the resources needed to accomplish it. The majority of the studies are restricted to traditional outcomes such as length of stay (LOS), mortality, and surgical complication rates. More recently, studies have considered using patient-reported outcomes measures (PROMs) and system-wide cost savings,^9^ which have increased the complexity of effectiveness analysis of service redesign.^10^ One recent study defined other relevant outcomes to the patient: the difference between quality and harm/safety and costs when assessing an ERP initiative.^11^ This adaptation of the value equation was conceived as the sum of patient-borne, third-party payer, and institutional costs.^11^

There are several existing regional and global initiatives to standardize outcomes evaluation by defining standard sets of measures for several health-care fields, such as the International Consortium for Health Outcomes Measurement (ICHOM), the Meetbaar Beter – Netherlands, the consolidated databases from Medicare, Medicaid and the Navy in the United States, and the National Health Service in the United Kingdom. The ERP guidelines usually include measures for health status achieved and recovery process that may be monitored for each patient.^5^ However, recommendations for standard methods to evaluate ERP implementation costs do not seem to be precise. In a systematic review that investigated the use of cost methods in the value-based health-care context, it was suggested that the methods currently applied to evaluate costs do not generate accurate cost information and, as a result, may have a reduced capability to be compared to or be used by future high-quality value analyses.^10^ A more recent study indicated a need to improve the definition of standard methods to correctly evaluate the costs associated with ERP adoption and improve the effectiveness analyses of ERP programs.^9^

Advanced cost accounting methods such as Time-Driven Activity-Based Costing^12^ (TDABC) have demonstrated the ability to measure costs with high accuracy and identify processes that can target quality improvement interventions in surgical pathways.^13–15^ For example, in total joint arthroplasty, it has been demonstrated that this method provided a more accurate measure of resource consumption than the traditional accounting methods generally used in hospitals. A recent publication by the Society for Perioperative Assessment and Quality Improvement suggested that TDABC costing^12^ as a methodology can help reduce inaccurate costing based on siloed budgets.^14^ The Society established eight consensus-based recommendations to apply TDABC to increase value in health care,^14^ and recently a TDABC in Healthcare Consortium (http://www.tdabcconsortium.com/) was created to bring together the expertise to clarify TDABC frameworks further and help with its dissemination.^16^

Beyond cost-saving opportunities with surgical redesign initiatives and ERP adoption, there is a potential to generate significant economic returns^17^ and optimize spending within complex health systems, such as the military and other siloed health organizations.^18^ To estimate these economic gains, it is necessary to consider the cost of the initial investment, establish data collection practices that allow an understanding of the patients’ journey over their care pathway, and use accurate financial methods to evaluate the return on investment.^17^ As we move toward a new era in the delivery of perioperative care, the adoption of programs that can improve the quality of care is of significant interest. However, there are many barriers to overcome before a comprehensive implementation is possible, especially considering the differences in health systems in high- and middle-income countries.^19^

One of the primary goals of the ERP care-design initiatives has been to center the delivery of care on the patient and improve the health-care value delivered.^20–22^ A standardized framework is required to guide the economic evaluation of such initiatives. Considering the need to overcome the differences between health systems and between the perioperative pathways, the TDABC is proposed as a template to provide additional tools to those who want to add value to the perioperative process.

The objective of this Recommendations Statement is twofold: first, to describe techniques and methods for applying each TDABC framework step; and second, to create a list of practice recommendations for how one could use the framework to support ERP development and associated decision-making processes.

## METHODOLOGY

**Figure 1.**
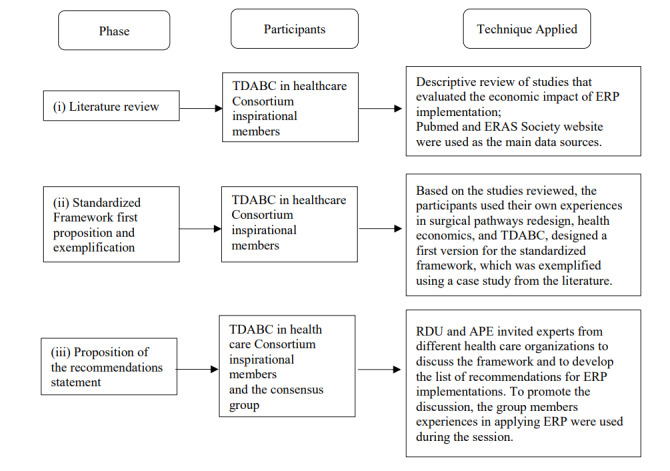
A Summary of the Methodology and Activities for the Recommendations from the Expert Group Abbreviations: APE, Ana Paula Etges; ERP, enhanced recovery pathway; ERAS, enhanced recovery after surgery; RU, Richard D. Urman; TDABC, time-driven activity-based costing.

The proposed standardized framework and the list of recommendations are based on the consensus group members’ current literature review and expert opinion. **Figure 1** shows the methodological sequence of the consensus process, which is comprised of three phases.

For the first phase, we searched Medline/Pubmed for applied case studies that evaluated the economic impact of ERP implementation. To guide the information extraction from those studies, we used the variable extractions of the following items: surgery type, patients’ clinical outcomes, the method used to evaluate costs, reduction in complication rates, LOS reduction, the report of cost savings, and, if available, information about how the ERP contributed to increasing health-care value. Descriptive analysis with a significant focus on the cost methods and economical and value assessments was used to report literature review results.

Consequently, in the second phase, members from the TDABC in Healthcare Consortium conducted a critical analysis of how TDABC can be implemented as a better cost method to guide the financial analysis of ERP implementations. One of the primary goals was to introduce a step-by-step standard framework to support the decision-making process before and during ERP pathway implementation. Our proposed framework is followed by a list of recommendations of how to apply each step.

In the third phase, a group of experts with doctoral-level education or significant experience in the surgical field’s health economics was invited for a focus group discussion. The session was moderated by two of the senior authors (APE and RDU) to discuss the steps of the framework and their applicability to health-care leaders. The moderators shared the article draft and the framework with the group of experts. During the focus group workshop, each member was asked about their agreement with each step. If a member identified a disagreement between one step, the moderators asked for the focus group member(s) to report an example from their real-world experience. After discussion designed to understand the example better, the group achieved a consensus. At the end of the focus group session, all the participants were invited to share their opinions about how the framework contributes to guiding ERP incorporation decisions and should be used in the health-care setting. These two final questions were essential before beginning work on the list of recommendations.

After the session, APE and RDU included the group’s recommendations for the framework and finalized the recommendations list designed to guide ERP design and implementation decisions. A first version of the list of recommendations was circulated among the consensus group members to review and validate each recommendation.

Finally, APE and RDU shared the participants’ final recommendations list, and the group reached a consensus regarding the importance of each item until the last Recommendations Statement was composed.

### The Cost and Economic Evaluation Process in ERP and Other Perioperative Surgical Pathway Redesign Implementations

Financial benefits associated with ERP implementation have been demonstrated in several procedures involving different surgical complexities and potential complications or adverse outcomes.^17,23^ Mastectomy surgery was the focus of a study that estimated a possible increase in revenue if the reduction in the LOS due to the adoption of the ERP protocol is considered in future mastectomy surgeries.^24^ In one gastroesophageal surgery study, cost savings associated with decreased length of time spent by patients in the intensive care unit were estimated.^25^ With a similar approach, Bisch et al.^26^ demonstrated the potential economic impact achieved by an ERP implementation for onco-gynecologic surgeries, by decreasing the LOS from 4 to 3 days and complications from 53% to 36%, with the net cost savings estimated at $936 per patient. More recently, outcomes and charges data from patients who underwent a redesigned pancreatic surgery pathway at MD Anderson Cancer Center were compared with US national administrative databases. The study demonstrated that the LOS and costs were reduced with ERP implementation by 1 day and 10%, respectively.^8^ However, the aforementioned studies used only hospital charges to predict or evaluate the economic impact of perioperative redesign. Detailed cost analyses considering advanced cost methods or real financial performance of hospitals were not conducted, but the study authors consistently underscored such approaches as future research endeavors.

Furthermore, another study focused on gynecologic procedures that applied a more rigorous cost analysis method to evaluate data on 1191 patients, comparing the costs of ERP and non-ERP patients—including labor, medication, materials, and hospital structure—based on data monitored and provided by the hospital finance department. This study also demonstrated a 7.8% decrease in LOS and 8.4% in costs per patient.^27^ Considering an indirect economic impact analysis, yet another study used data provided by the finance department to estimate the indirect gains obtained by the reduction of materials and medications consumed and the length of time spent by patients in intensive care units as the result of the adoption of ERP protocols for bariatric surgery.^28^

One recently published study^29^ applied the TDABC framework to evaluate the cost-effectiveness of microvascular breast surgeries. The use of the method allowed more detailed cost accounting and data accuracy of direct and indirect costs by enabling patient-specific resource consumption over the care pathway.^30^ This method has been applied successfully as a micro-costing technique in medical research,^31^ suggested in the literature as a useful method for initiatives seeking value-based analysis.^31,32^ Since the microvascular breast surgery studies focused on cost-effectiveness, the TDABC was particularly well-suited to evaluate the required processes redesign to implement the ERP. The study also pointed out as a benefit of the method its capability to generate a shared understanding among clinicians and administrators of the direct costs incurred due to ERP implementation, while fostering the ability to benchmark costs across institutions.

Many health-care organizations have successfully implemented ERP protocols and conducted surgical care redesign initiatives often based on professional society recommendations, such as those of the Enhanced Recovery After Surgery Society (https://erassociety.org/) and the American Society for Enhanced Recovery (http://www.aserhq.org/web/). At Dell Medical School at the University of Texas at Austin, a group of researchers applied the re-engineering and design thinking methods in an internal program, “Preoperative Assessment and Global Optimization.”^33,34^ This program encompasses the entire episode of care and is designed to guide patients and their family members through the complexities across the perioperative pathway. It emphasizes patient-centered care, shared decision-making, rigorous process standardization, the use of evidence-based clinical care pathways to achieve current best practices, and robust coordination and integration of care. For the researchers, the program represents an evolution and consolidation of several existing system-wide Preadmission Testing Clinics into a more efficient “hub-and-spoke” model. However, economic analyses for this model are yet to be developed.

The assumption that clinical and associated financial outcomes will accrue with minimal investment^17^ seems to be a common mistake since this variable is excluded from the studies, and financial outcomes generally estimated by future cost savings are being calculated with only secondary data. Measuring outcomes that matter to patients and affect costs are elements of the Value Agenda.^6^ The consideration of a standardized framework to guide health-care managers and decision-makers to evaluate the actual economic impact associated with a perioperative pathway redesign can allow the value measurement achieved in each initiative with more accuracy.

### The Standardized Framework To Guide the Decision-making Process of ERP Incorporation into the Clinical Setting

**Figure 2.**
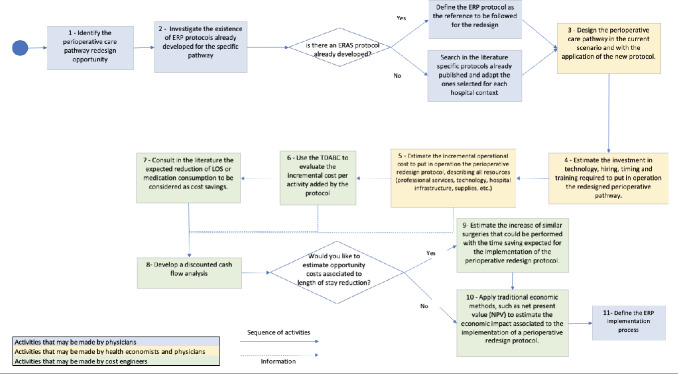
Standardized Framework To Guide the ERP Incorporation into the Decision-making Process

Understanding the financial implications of ERP requires an understanding of the investments needed for successful implementation and the detailing of cost savings that can accrue when an ERP has been fully incorporated into clinical practice.^17^ A combination of traditional economic analysis methods, such as Net Present Value (NPV) and TDABC to estimate cost savings (considered revenue for economic analysis), can evaluate the real economic impact of the perioperative pathway redesign. The NPV results from the difference between all future cash flows over the entire life of an investment discounted to the present.^35^ To calculate the NPV, it is necessary to analyze in a discounted cash flow the investment required to successfully implement the redesigned perioperative pathway and the expected cost savings over the years, and to define a period to analyze the economic impact. The flow chart shows the details of how those methods can be applied in a pathway redesign initiative (**Figure 2**).

This framework guides the analysis that should be performed before the decision to implement an ERP is taken, suggests the economic impact that the ERP implementation can achieve, and serves as a basis for administrators and clinicians to discuss the redesign opportunity in detail. By detailing the care pathways in a TDABC study, the multidisciplinary teams can also learn in detail the sequence of activities related to patient care and all resources needed to perform those activities.^36^ Multidisciplinary process mapping sessions, capacity-cost calculations, and model integration were coordinated and offered to engage care providers at each phase. Once the TDABC is used in the process of an ERP implementation, all the additional resources that may be needed to implement the ERP are identified. By generating these information resources and cost estimates, the risk of starting a project without having all the resources necessary to make it successful can be mitigated. We believe that this approach can facilitate engaging the entire clinical team during a particular redesign initiative.

The consensus group developed a detailed list of recommendations to be followed in each step during the implementation process. We outline 11 key steps below:

### Step 1: Identify the Perioperative Care Pathway Redesign Opportunity

For the redesign project to fit given value-based health-care goals, it is suggested that the care pathway selection that will have a redesign evaluation consider patient volume at the institution that will be subjected to the care pathway and the clinical literature available indicating that it is possible to achieve better health outcomes by designing a given ERP.^37^ The high volume suggests that the clinical team has a high level of experience in the specific procedures that will be studied to define the redesign standards of care and also contributes to the achievement of the financial results faster. Additionally, the clinical team should demonstrate an interest in collaborating to redesign their current practices of providing care to patients.

### Step 2: Investigate the Existence of ERP Protocols Already Developed for the Specific Pathway

Once the focus of a redesign surgical pathway is defined, the first activity should examine published protocols in the existing literature. If the hospital already has other ERP protocols implemented and a specific protocol for this surgery that a professional society has endorsed, the ERP protocol can be considered the desired clinical practice. However, if the hospital has not yet implemented ERP protocols and there is no specific protocol for the surgery care pathway under evaluation, a literature review to identify best practices that can support the ERP design may be considered.

### Step 3: Design the Perioperative Care Pathway in the Current Scenario and with the Application of the New Protocol

Design thinking and the Business Process Model and Notation^38^ can be considered as implementation techniques to describe the current practice of providing care to patients and how it needs to be modified to execute the new protocol. The clinical team’s involvement in this phase is essential for achieving a care map representing the real-world setting and can better indicate how the new protocol will work in the institution.

### Step 4: Estimate the Investment in Technology, Personnel, Timing, and Training Required To Set Up the Redesigned Perioperative Pathway

All the required investments needed to operationalize the perioperative care pathway should be identified and budgeted. The technology may be described as medical equipment, telemedicine platforms, additional data cloud storage, software, or app development, if necessary. A detailed classification of the job descriptions can be developed for all the clinical and non-clinical staff for hire. The remuneration can be estimated according to the job description if it is necessary to hire new professionals. An education program to engage the team on the redesigned processes can be organized according to the estimated schedule for starting the redesigned perioperative care pathway. It is also essential to calculate the expected timing to structure the redesigned care pathway and, if necessary, a team to structure it (including clinician and non-clinician hours).

### Step 5: Estimate the Incremental Operational Cost To Set Up (or to establish) the ERP, Describing All Resources (professional services, technology, hospital infrastructure, supplies, etc.)

For the designed ERP, all the additional resources necessary to perform each activity should be identified. The resources can be divided into technology, professional services, supplies, and hospital infrastructure. For example, technology may represent telemedicine services or innovative equipment necessary along the care pathway; professional services include all possible employees who dedicate additional time to the patients under ERP protocols. Changing postoperative patient allocation should also be considered, as in intensive care unit or high-dependency unit allocation for high-risk surgical patients or a hospital ward dedicated to hip surgery patients. The sum of additional resources identified represents the incremental operational cost of the ERP.

### Step 6: Use of TDABC To Evaluate the Incremental Cost per Activity Added by the Protocol

The methodological sequence of steps to apply the TDABC in health care should be followed.^39^ The time and financial data collection deserve particular attention from the project executors to achieve accurate cost information with TDABC.^16^ The involvement of financial and internal management and operations departments is essential to estimate hospital structural and labor costs. Regarding time data collection, considering that the new care pathway may not yet be in place, it is suggested that the clinicians offer an informed estimation of the length of time for the sequence of activities indicated in the clinical protocol. For the existing care pathway, once the ERP is implemented, the time data should be reviewed by doing chrono-analysis studies.

Once the cost-per-activity for the two care pathways is calculated, the cost results can be compared and the differences observed for each activity and the care episode may be considered potential incremental cost, or cost-savings.

### Step 7: Literature Search Regarding the Expected Reduction of LOS and Readmission Rates

Previous literature associated with the ERP protocol being implemented is the primary reference for the anticipated impact on patient LOS and health-care services consumption. However, after implementing the ERP, the mean LOS and readmission rates with the real-world data registered at the institution should be reviewed.

### Step 8: Develop a Discounted Cash Flow Analysis

After the total investment, incremental costs, and potential cost-savings are calculated, it is necessary to preview the volume of patients expected to undergo the procedure over a period of time and assume a weighted average cost of capital according to the institutional capital cost. A discounted cash flow can then be structured, and the NPV calculated.

A hypothetical example can elucidate this process. An investment need of US$30 000 was estimated to implement a perioperative care pathway; the hospital established a team of anesthesiologists and surgeons to support the program, composed of physicians from the hospital who had their current practices redesigned, and these resulted in an incremental service cost of US$9675. A mean decrease of 2 days of the hospital LOS per patient was observed. For this example, a Medicare hospitalization charge per day was considered. Therefore, assuming a weighted average cost of capital of 5% per year in hospital practices (0.41% per month) and an average of 10 patients undergoing the care pathway per month, the protocol implementation could represent an NPV of US$80 910 in 6 months.

The economic analysis could also include other cost-saving or cost-increase variables, if identified later, such as a decrease in medication consumption or additional costs related to complication rates or other outcomes such as unplanned intensive care unit admissions, surgical re-interventions, or postoperative death.

### Steps 9 and Step 10: Search for Additional Opportunity Costs

These two final steps are optional, but are recommended for hospital leaders who wish to incorporate opportunity costs associated with decreasing patient LOS into the economic analysis. In this scenario, hospital leaders would estimate the different surgeries that could be done within the exact structure of the surgical department, per period of analysis, and add their financial results as financial gains to the discounted cash flow. If the leaders identify that there is no pent-up demand for any procedure, another way to include the opportunity cost associated with the decrease in LOS is to evaluate a project to reduce the operating room capacity and make it less idle.

Complementary outcomes to be evaluated can be chosen according to the previous discussion with peers and stakeholders. For example, if the institution is organized to collect and manage patient-reported outcomes or patient-reported experience, given some evidence of their improvement with ERP, these must be considered in new pragmatic studies.^40,41^

The NPV may be recalculated to include the extra financial gain from the additional procedures that can be performed within the same operating room structure. Finally, improvement in patient experience scores reported by patient-reported outcomes and patient-reported experience measures may also be evaluated, and radar charts with value measures can be used to express the gains.^8,42^

### Step 11: Define the ERP Implementation Process

Implementing a perioperative care pathway as a quality improvement project may pose numerous operational challenges. After the planning process, to successfully implement any change some important principles should be followed: (i) get support from all stakeholders; (ii) form an implementation team with the hospital quality improvement leaders, the recovery room and ward nurse leaders, and the perioperative anesthetists and surgical teams; (iii) adopt an established quality improvement model such as one based on “plan-do-check-act” cycles across the phases of the perioperative pathway; (iv) engage surgical teams in adopting the postoperative bundle within their assistance; and (v) audit perioperative practices to examine the new protocol compliance and keep perioperative caregivers updated.

### The Introduction of the List of Recommendations

The framework described in this Recommendations Statements—building on the suggestions presented by Najjar et al.^17^—seeks to complement detailed economic analytic methods in supporting decision-making processes related to an ERP implementation. Since protocols are usually composed of activities distributed throughout the patient care pathway, TDABC may be valuable in evaluating the net costs associated with an ERP protocol implementation.

With millions of surgeries performed annually in the United States, an estimated 51% of the total Medicare expenses are consumed by surgical care, and these expenditures continue to rise.^43^ From 2005 to 2025, the aggregate surgical expenses will increase by approximately 100%.^44^ By understanding the burden of surgical episodes in the health system, reimbursement and ERP designs have been introduced to reduce health-care costs (and waste) and improve the quality of care.^4^ However, the cost of surgical care is currently poorly understood.^43^ The framework introduced here can guide decisions in an era where *how* things are being done is just as important as *what* it is being done. In emerging economies where the administrative and patient outcomes data availability is limited, the redesign of surgical pathways can be facilitated by following the proposed step-by-step process.

Once the perioperative redesign protocols begin to include robust economic analyses, it will be possible to measure the resulting value expansion opportunities. Given how the impact of the implementation of protocols on functional and patient-reported outcomes is already known and available in the literature,^2,5^ the adoption of detailed economic analyses will allow the value equation to be applied in the real world.

One of the LEAN management techniques recommended by the value-based health-care implementation literature as a data-driven strategy to increase health-care efficiency is the Define Measure Analyze Improve and Control (DMAIC) method.^28,45^ A systematic review published in 2015 identified 23 applied studies in the surgical field, with 88% of them demonstrating positive results such as cost reduction and quality improvement.^46^ One notable example is the study conducted in Italy showing a 42% reduction in LOS.^47^

**Figure 3.**
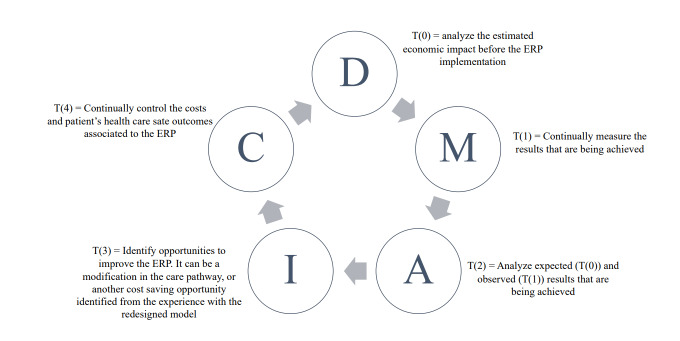
DMAIC and the ERP Framework The figure describes how the DMAIC method can be combined to the ERP framework introduced in optimizing the decision-making process of the ERP implementation and in operationalizing the ERP management process.

The standardized framework to guide the decision-making process of ERP incorporation can help those involved in care redesign follow the DMAIC cycle. We understand that the latter framework can be used before the ERP incorporation by supporting the definition of the project and measuring its potential impact. Once the decision is made to incorporate the ERP design project, the economic steps (4 to 10) listed in the framework can be continuously monitored to assess the real economic impact and identify improvement opportunities by analyzing the observed results. **Figure 3** suggests how the framework introduced in this article is related to the DMAIC continuous improvement cycle.

The framework introduced in this Recommendations Statement might provide health-care administrators with the ability to generate information for value equation variables^11^ to support the decision-making process of incorporating an ERP. Additionally, the use of TDABC to measure financial and time of care process outcomes for health-care redesign initiatives represents innovation and one more value attribute of TDABC for health-care management.

Based on the expert consensus framework introduced here and the previous recommendations for using TDABC to evaluate and identify value increase opportunities in surgical settings,^14^ the TDABC in Healthcare Consortium presents a list of six recommendations for using our Standardized Framework to guide the decision-making processes of ERP incorporation and management. The significance of our proposed framework is that it provides recommendations that define how the TDABC can be used to support the decision-making process to incorporate and manage surgical pathways.

### Recommendations for Using the Proposed ERP Framework

1. Use the ERP framework to guide the ERP design decisions. By applying the framework, it is possible to estimate the economic impact of the surgical pathway redesign.
2. Educate health-care professionals about the designed ERP. Applying the framework, including the TDABC methodology, requires a multidisciplinary team approach, helping clinicians learn about finance and the administrators and financial analysts to learn about clinical care pathways.
3. Support the continuous improvement cycle. Applying the proposed framework with the DMAIC cycle in mind and involving multidisciplinary groups can help identify opportunities to make the care cycle more efficient.
4. Minimize unnecessary variability and move toward care standardization. By applying the TDABC methodology and detailing care cycles that identify the resources necessary to provide high-quality care to patients, standards of care are defined.
5. Utilize data to monitor the pathway financial results continuously. Once the framework is applied to support the redesign incorporation decision, it must be frequently updated in a structured manner to provide real-world data to administrators and guide economic choices that increase the value delivered through the surgical pathways.
6. Utilize the framework to bring internal and external transparency to the management of surgical pathways, especially in middle-income countries where databases that collect outcomes are beginning to be utilized. Share the results with payers and internal teams, highlighting the benchmarks identified and encourage them to achieve better health outcomes and economic impacts.

### Limitations and Future Research Direction

While comprehensive, the framework has a few limitations. First, we did not develop a new systematic review to evaluate all studies that developed economic methods to evaluate ERP implementations. We used recent review articles published to sustain our justifications and economic methods selected to build the framework,^9,10,15^ and we believe that our reliance on our and others’ prior research formed a methodologically robust basis for the recommendation statements. We also did not evaluate any prioritization between the different recommendations, which can be better studied in future applied analyses. It is essential to highlight that using the TDABC approach to sustain the decision-making process to incorporate surgical ERP may be accompanied by instruments to evaluate recovery and clinical outcomes because this approach can enable a better understanding of the cost impact, however, it does not include any health status outcome measures. Finally, this is ultimately a theoretical proposition, and we recommend evaluating the value of its application in future case studies.
